# The Hypophosphites—Their Therapeutic Value

**Published:** 1866-05

**Authors:** Ira D. Brown

**Affiliations:** Resident Physician and Surgeon of Albany City Hospital


*The Hypophosphites—Their Therapeutic Value.*—By IRA D. BROWN, M. D., Resident Physician and Surgeon of Albany City Hospital.

It must be confessed by all candid and reflecting physicians that the medical profession is exceedingly slow to learn new facts. Such is the reluctance to forsake old methods of practice, that many stumble heedlessly on through life without once getting out of the rut in which they started. With these, inflammation is still the *phlogiston* of the ancients, and a heated surface and bounding pulse the signal for depletion, antimony, calomel, jalap, &c., without regard to time, place or circumstances. Such men seldom learn anything—never make discoveries—never accomplish anything worthy of the noble profession in which they stand immovable as lamp-posts, bearing up only the light of others. On the other hand, there have always been men in the profession who are but too ready to experiment—who, having no stable theory of their own, eagerly embrace the last one that is presented. These are victims in every turn of new delusion; they are ever astride of some hobby, from which they fall only to mount another.

The first class named will always remain obstinate stumbling blocks in the path of scientific progress; truth must advance directly over them, if it advance at all. The second class are the fruitful parents of homeopathy, hydropathy, and kindred shams which, at various periods, have scourged the world and contributed greatly to bring the healing art into disgrace.

We have—thank heaven!—a third class of physicians, never too old or too wise to learn, who are willing to give any theory a fair investigation—to accept whatever of truth may be found in it, and reject with keen discrimination that which stands upon untenable foundations. These are the *real*, the *true* physicians of the world, and worthy to stand by the bedside and minister to the ailments of suffering humanity. They are the men who make discoveries—who do not deem it labor lost to look through a bushel of the chaff of medical literature if they can separate from it one grain of truth—who are willing to mine patiently for the diamond *fact*, though it be embedded never so deep in the clay of absurdity, speculation and folly.

When, less than ten years ago, Dr. J. Francis Churchill announced to the Imperial Academy at Paris that he had discovered a remedy for pulmonary phthisis, the first class of doctors I have referred to simply pronounced the statement absurd, and continued to stumble along in the old paths with which they were familiar. The second class (such of them as chanced at the time to be unhorsed from some of their previous hobbies) mounted Dr.

Churchill's theory and well nigh rode it to death, carrying it to the most ridiculous extremes. The result has been, however, that some of the third class of quiet, thinking students of their profession, having applied the tests of judgment and experience to the subject, have found a new and important agent in the materia medica, which, if not equal to all the sanguine anticipations of Dr. Churchill, is still of permanent therapeutic value.

As is well known to those who have read the work of Dr. Churchill, which must ever remain a lasting monument of the enthusiastic research of that physician, the remedies on which he mainly relies are the hypophosphite salts resulting from a union of hypophosphorous acid with a salifiable base. Dr. Churchill's investigations led him to the conclusion that the tubercular diathesis depended upon a deficiency of oxidizable phosphorous in the system, and that the appropriate remedy would be the exhibition of phosphorus in a form capable of oxidation and assimilation. That phosphorus exists in large proportion in the animal economy has long been known, and the additional fact that it is found free in the brain is a demonstration of modern chemieal investigation. It cannot be doubted by any intelligent mind that the phosphorus present in the body performs *some* important office, for nature does not distribute the elements without an object. In an article published in *The Boston Medical and Surgical Journal*, in May, 1858, Dr. Nichols, the well known chemist, remarks: "The vital importance of these agents (the hypophosphites) in maintaining a normal condition of the system can be understood by a consideration of the probable fact that in all the operations of the mind, in every effort requiring an expenditure of nervous force, they are called into action. In their rapid oxidation in the brain, on occasions of great intellectual effort, there may be a nearer approximation to literal truth in the remark that there are 'thoughts that burn,' than is generally supposed." This observation is supported by the fact that students and others who are performing a large amount of brain labor excrete an unusual amount of phosphates in the urine, a circumstance I have had the curiosity to test repeatedly. It has been asserted—I know not with what truth—that there is an almost total want of phosphorus in the brains of idiots. If chemical science should succeed in demonstrating this circumstance, it would add another convincing proof of the importance of this element as a generator of nervous power, and might afford a useful hint for the treatment of insanity and other mental aberrations.

It may be assumed, without any stretch of rational conclusion, that if oxidizable phosphorus exists in the brain, blood and tissues of the animal body in its normal condition, its absence or diminution must create variations from the healthy standard—in a word, *disease.* It thence irresistibly follows that the remedy consists in supplying the deficient element in a condition both assimilable and oxidizable. There is no dispute among chemists or physiologists as to the fact that the hypophosphite salts offer the most direct and philosophical means of supplying phosphorus to the system. The small amount of oxygen in combination renders them easily decomposable in the economy. In the language of Dr. Nichols, "what phosphoric acid may have failed to do in supplying the waste of phosphorus, it is almost certain that the acid containing the less amount of oxygen is capable of accomplishing." The principal theory, then, for the administration of the hypophosphites, rests upon these three foundations:

1. That phosphorus is an essential element of the animal economy.
2. That its undue waste by excretion, or otherwise, is a cause of disease.
3. That the natural remedy is phosphorus in an oxidizable and assimilable form.

That the hypophosphites possess alterative and tonic properties cannot be doubted by any person who has witnessed the sometimes surprising effects following their administration. Dr. Newton says of them, in the *Chem. Gazette*, "They seem to possess the power of increasing nerve force and promoting the function of nutrition." Another effect which has often been noticed by those who are familiar with their administration, is the apparently anodyne influence exerted in cases of morbid vigilance and restlessness. Although the patient may have been disturbed and wakeful, no sooner does he commence taking the hyopphosphites than he sleeps soundly at night. They produce no headache, constipation, or other unpleasant symptom, and the patient does not ordinarily become accustomed to them so as to require an increase of dose. The anodyne effect is probably incidental, and follows from the tonic impression upon the digestive apparatus, as it is well known that judicious physical exercise, or anything that improves the assimilation of the food, produces sleep in the same way.

While it yet remains to be proved that any remedy is capable of curing a well-marked case of phthisis that has advanced to the second stage, there can be no reasonable doubt that the hypophosphites, beyond any other remedy that we possess, will prolong the life of the patient. Dr. Wood believes that consumption is occasionally curable. That the disease depends chiefly upon defective nutrition, is almost universally admitted, and if any medicine is capable of remedying the imperfect assimilation of the food, the hypophosphites will accomplish it, for therein seems to lie their peculiar power. I have seen one case where the patient was much reduced in flesh and strength, with night-sweats and a racking cough, apparently dependent upon tubercular deposit in the left lung, which resulted favorably. Under the use of the hypophosphites, the patient gained strength daily, the night-sweats ceased, appetite returned, and he got rapidly well. I saw the patient again twelve months afterwards, and he appeared to be enjoying very tolerable health, without any signs of the disease which had threatened to put a speedy end to his existence. In the first, or forming stage of phthisis, the hypophosphites may be given with every hope of a favorable result.

But if Dr. Churchill did not discover a remedy for tuberculosis, he certainly did bring before the medical world a class of medicines that are of the greatest value in the numerous diseases resulting from the loss of nerve power; also in many of the diseases of infancy connected with the scrofulous diathesis, and those where the osseous system is defective. In all these cases I have seen them administered with the most beneficial results, and will here mention two or three cases as illustrations.

Mrs. N., 34 years of age, married, of delicate nervous organization; had suffered much from dismenorrhœa; never had children. Patient, when she came under my notice, was considerably emaciated; countenance pale; pulse weak and considerably accelerated; much troubled with cold hands and feet; appetite capricious, but generally poor; frequent pain and sickness at stomach after meals; has some cough; is dejected and gloomy in spirits; had taken elixir of bark and iron, quinine, cod-liver oil, and various other tonics, with very little benefit. Prescribed the following: ℞ Hypophosphite lime, hypophosphite soda, āā ʒij.; water, Oj. A tablespoonful to be taken thrice daily. Without other treatment the patient rapidly recovered her health and spirits; appetite became excellent; digestion good; gained several pounds in weight, and after taking the hypophosphites six weeks, was to all appearance perfectly well.

Mrs. H., aged 29, had enjoyed very good health until after the birth of her second child, after which she suffered for a year with indigestion, and the long train of nervous symptoms so often witnessed by practitioners that they scarcely need description. She became a prey to the most dismal fancies and gloomy forebodings; passed whole nights without sleep, often in paroxysms of mental anguish distressing to witness. Opiates seemed only to increase the sleeplessness of the patient. She was treated in turn by two medical gentlemen, who exhausted the whole catalogue of tonics and antispasmodics without giving any relief. When I saw the patient, in addition to the symptoms above detailed, there was much tenderness perceptible upon pressing the fingers on the upper part of the cervical vertebræ. She was ordered to take ten grains of the hypophosphite of lime three times a day. No other treatment was had, with the exception that the patient was directed at first to take a dose of ammoniated tincture of valerian at bedtime, which was soon discontinued. Improvement was noticed almost immediately. On the second night her sleep was undisturbed. After continuing the medicine nearly two months, she appeared perfectly well; appetite and digestion good; sleep sound and refreshing. She assured me that she could never be sufficiently grateful for recovery from a condition she said was "worse than death."

I have only time and space to give one more case, which was that of a boy three years of age, suffering from marasmus. The little patient was much emaciated, his abdomen was distended, he had a diarrhœa, which his mother said had continued with occasional intermissions for nearly two years, sometimes accompanied with bloody discharges. Under the administration of the hypophosphites, taken in milk, two grains a day, his recovery, apparently permanent, took place in a few weeks. I have known a somewhat similar case recover under the use of the syrup of the pyrophosphate of iron, and will not stop now to consider whether the iron or phosphorus, or both, cured the patient.

Without protracting the discussion further, have I not said enough to warrant the conclusion that the hypophosphites are worthy a more prominent place than the appendix to the United States Dispensatory? In the class of diseases to which they seem so well adapted, I believe they will disappoint the practitioner in fewer instances than more pretentious remedies that come to us labelled *officinal.*

It may be proper to add, by way of caution to those who may desire to make a trial of the hypophosphites, that some of these salts in the market I have found nearly worthless, they doubtless being improperly prepared. It is of the utmost importance that they should be pure, containing the proper proportion of hypophosporic acid, and it will be well to see that they come from the laboratory of some reliable chemist.—*Boston Medical and Surgical Journal.*

